# Monosomy 3 Is Linked to Resistance to MEK Inhibitors in Uveal Melanoma

**DOI:** 10.3390/ijms22136727

**Published:** 2021-06-23

**Authors:** Svenja Mergener, Jens T. Siveke, Samuel Peña-Llopis

**Affiliations:** 1 Translational Genomics in Solid Tumors, German Cancer Consortium (DKTK) and German Cancer Research Center (DKFZ), University Hospital Essen, Hufelandstrasse 55, D-45147 Essen, Germany; s.mergener@dkfz.de; 2 Division of Solid Tumor Translational Oncology, German Cancer Consortium (DKTK) and German Cancer Research Center (DKFZ), D-69120 Heidelberg, Germany; j.siveke@dkfz-heidelberg.de; 3 Bridge Institute of Experimental Tumor Therapy, West German Cancer Center, University Hospital Essen, Hufelandstrasse 55, D-45147 Essen, Germany

**Keywords:** ocular melanoma, monosomy 3, targeted therapy, MAPK, BRCA1-associated protein 1, eIF2 signaling, eIF2alpha

## Abstract

The use of MEK inhibitors in the therapy of uveal melanoma (UM) has been investigated widely but has failed to show benefits in clinical trials due to fast acquisition of resistance. In this study, we investigated a variety of therapeutic compounds in primary-derived uveal melanoma cell lines and found monosomy of chromosome 3 (M3) and mutations in *BAP1* to be associated with higher resistance to MEK inhibition. However, reconstitution of *BAP1* in a BAP1-deficient UM cell line was unable to restore sensitivity to MEK inhibition. We then compared UM tumors from The Cancer Genome Atlas (TCGA) with mutations in *BAP1* with tumors with wild-type *BAP1*. Principal component analysis (PCA) clearly differentiated both groups of tumors, which displayed disparate overall and progression-free survival data. Further analysis provided insight into differential expression of genes involved in signaling pathways, suggesting that the downregulation of the eukaryotic translation initiation factor 2A (*EIF2A*) observed in UM tumors with *BAP1* mutations and M3 UM cell lines might lead to a decrease in ribosome biogenesis while inducing an adaptive response to stress. Taken together, our study links loss of chromosome 3 with decreased sensitivity to MEK inhibition and gives insight into possible related mechanisms, whose understanding is fundamental to overcome resistance in this aggressive tumor.

## 1. Introduction

Uveal melanoma (UM) is the most common malignancy of the eye, affecting 5 to 7 individuals per million people per year [1]. While primary tumors are usually treated successfully by surgical resection or radiotherapy, around 50% of patients develop metastatic disease within a few years after diagnosis [2]. Once metastasized, median survival is very low, ranging between 3.7 to 7 months, and has not changed during the past decades due to a persisting lack of effective treatment options [3,4,5,6]. Since UM is highly resistant to chemotherapy and immunotherapy-based approaches (reviewed in [7]), targeted therapeutic compounds specifically affecting dysregulated cancer-promoting molecular pathways could serve as promising alternatives. Targeted therapy has been proven beneficial in cancers with certain molecular changes resulting in a dependency on specific signaling pathways. This has been shown by successful treatment of endothelial growth factor receptor (*EGFR*)-mutated non-small cell lung cancer (NSCLC) with EGFR inhibitors, treatment of breast cancer gene (*BRCA*)-mutated ovarian cancer with poly-ADP (adenosine diphosphate)-ribose polymerase (PARP) inhibitors and treatment of *BRAF*-mutated cutaneous melanoma and NSCLC with BRAF inhibitors or MEK inhibitors [8,9,10,11]. However, in uveal melanoma, an extraordinarily low mutational burden complicates the identification of suitable targets [12].

Around 90% of UM tumors harbor mutually exclusive mutations in two homologous G-protein alpha subunits, *GNAQ* and *GNA11*, which are considered to be early events and key drivers of UM development and lead to a constitutive activation of the mitogen-activated protein kinase (MAPK)/ERK pathway [13,14,15]. Activation of the MAPK/ERK pathway is a well-described oncogenic event involving a cascade of consecutive activation of RAS, RAF and MEK resulting in activation of ERK, leading to cell proliferation and survival (reviewed in [16]). Since inhibition of the constitutive active MAPK/ERK pathway by targeting MEK in *BRAFV600E*-mutated cutaneous melanoma has been shown to be successful, MEK inhibitors have also been investigated as potential therapies in UM [17,18]. Despite promising preclinical results, clinical studies involving the MEK inhibitors selumetinib and trametinib failed to show any benefits in UM [19,20].

One of the most important prognostic factors in UM is monosomy of chromosome 3. Monosomy 3 (M3) is strongly correlated with metastatic risk, and gene clusters on chromosome 3 as well as on chromosome 8p build a basis for classification of UM tumors in a high or low risk class using microarray-based gene expression profiling [21,22,23]. Moreover, mutations in BRCA1-associated protein 1 *(BAP1)* have been found in more than 80% of metastasizing UM [24]. *BAP1* is a nuclear deubiquitinase which, besides its capacity of deubiquinating proteins, also functions as a central binding partner in complexes with BRCA1, HCF1 or ASXL1, among others, thereby affecting cell proliferation, DNA damage response and differentiation processes through influencing chromatin remodeling [25,26,27]. BAP1 functions as a tumor suppressor in mesothelioma, intrahepatic cholangiocarcinoma (ICC) and renal cell carcinoma (RCC) [28,29,30]. In UM, mutations in *BAP1* have been shown to promote a stem cell-like state and loss of protein expression has been associated with increased metastatic risk [31,32]. Since the *BAP1* gene is encoded on chromosome 3p.21, it is likely that M3 in UM unmasks recessive mutations in *BAP1* [24].

To the best of our knowledge, this study reports for the first time that patient-derived UM cell lines with M3 show increased resistance to inhibitors targeting MEK compared to disomy 3 (D3) UM cell lines. We placed these findings in the context of *BAP1* mutational status. However, reconstitution of wild-type *BAP1* in a BAP1-deficient M3 cell line was unable to sensitize cells to MEK inhibitors, suggesting that other mechanisms associated with loss of chromosome 3 might affect sensitivity to MEK inhibition in combination or independently of BAP1 loss. To address this, we performed an analysis of The Cancer Genome Atlas (TCGA) for UM to compare the pathways that are overrepresented in UM tumors with *BAP1* mutations and M3 compared to UM tumors with wild-type *BAP1* and D3. We identified eIF2 signaling and ERK/MAPK signaling as important pathways. These findings can help to build a path for understanding resistance mechanisms in UM, which is critical for learning how to effectively target this lethal cancer.

## 2. Results

### 2.1. Cell Lines with Monosomy 3 Show Higher Resistance to MEK Inhibition

**Figure 1:**
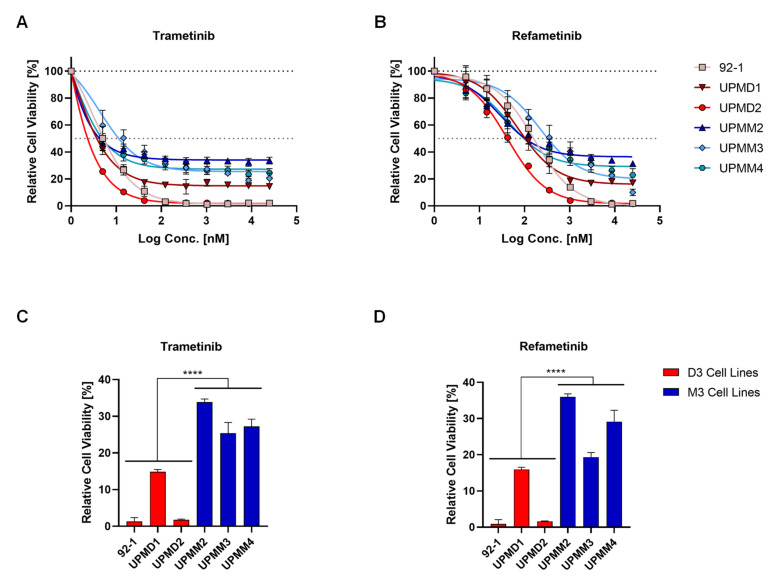
Uveal melanoma cell lines with monosomy 3 (M3) are resistant to MEK inhibition compared to cell lines with disomy 3 (D3). Dose-response curves of uveal melanoma cell lines with M3 (blue symbols) and D3 (red symbols) that were treated with trametinib (**A**) or refametinib (**B**) at different concentrations (from 5 nM to 25 µM) for 5 days and minimum cell viability of each cell line after treatment (**C**,**D**). Cell viability is expressed as normalized values in percentage to DMSO controls. Summarized results of three independent experiments are shown. Data are average ± SD. ****, *p* < 0.0001.

We evaluated the sensitivity of three D3 (92-1, UPMD1 and UPMD2) and three M3 (UPMM2, UPMM3 and UPMM4) UM cell lines to a variety of chemotherapeutic and molecularly targeted compounds at nine different concentrations to obtain comprehensive dose-response curves throughout different inhibitor classes. Treatment of UM cell lines with MEK inhibitors for 5 days resulted in a significantly higher cell viability for M3 cell lines than D3 cell lines after treatment, even after exposure to high concentrations (>20 µM) (Figure 1). Trametinib displayed strong potency in decreasing cell viability at low concentrations with a reduction of viable cells from 40% (UPMM4) to 70% (92-1) at 5 nM. However, effects reached a plateau at a remaining viability of around 30% to 40% in all M3 cell lines (Figure 1A). D3 cell lines 92-1 and UPMD2 can be classified as sensitive to MEK inhibition and only show minimal leftover cell viability at concentrations higher than 100 nM trametinib (Figure 1A) or 1 µM refametinib (Figure 1B). Cell viability in D3 cell line UPMD1 plateaus after initial reduction but is substantially lower than in M3 cell lines (Figure 1A–D). This resistance pattern of M3 cell lines to MEK inhibitors could not be observed after treatment with other compounds, such as the proteasomal inhibitor carfilzomib and the receptor tyrosine kinase inhibitor sunitinib (Supplementary Figure S1).

### 2.2. BAP1-Reconstitution in a M3 Cell Line Does Not Influence Sensitivity to MEKi

**Figure 2:**
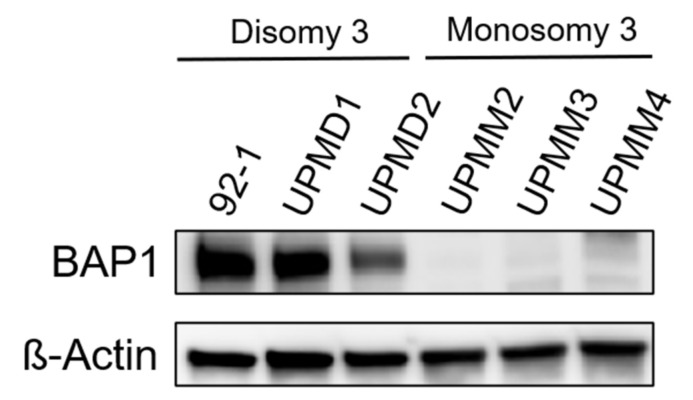
Uveal melanoma cell lines with monosomy 3 (M3) show BAP1 loss. BAP1 protein expression was assessed in the indicated uveal melanoma cell lines by Western blot. A representative result of three independent experiments is shown. Uncropped blots are displayed in Supplementary Figure S2.

**Figure 3:**
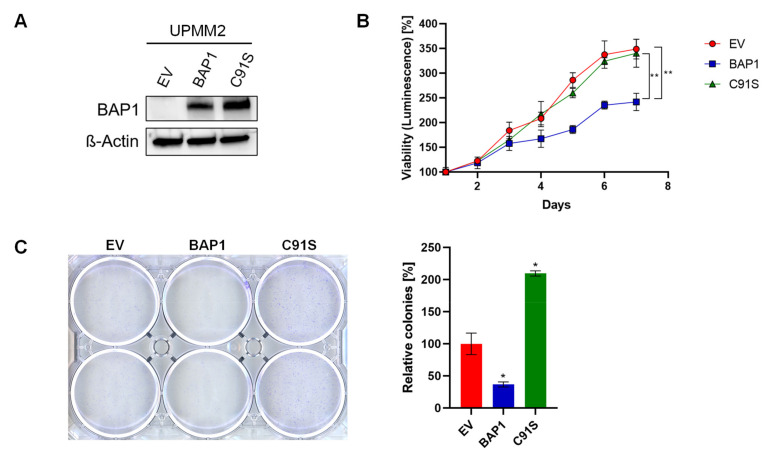
Establishment of a *BAP1*-reconstituted UM cell line. M3 cell line UPMM2 was reconstituted with an empty vector (EV), wild-type *BAP1* or an inactive p.C91S mutant *BAP1* variant and further characterized. (**A**) Western blot confirms protein expression of *BAP1* in *BAP1*-deficient cell line UPMM2 after wild-type or mutant *BAP1* reconstitution. (**B**) Reconstitution of wild-type *BAP1* in cell line UPMM2 leads to a significant decrease in proliferation rate. A representative result of three independent experiments is shown. (**C**) Reconstitution of wild-type BAP1 in cell line UPMM2 shows a tendency of less colony formation ability when seeded at low density. A representative result of three independent experiments is shown. Data are average ± SD. *, *p* < 0.05. **, *p* < 0.01. Uncropped blots are displayed in Supplementary Figure S3.

**Figure 4:**
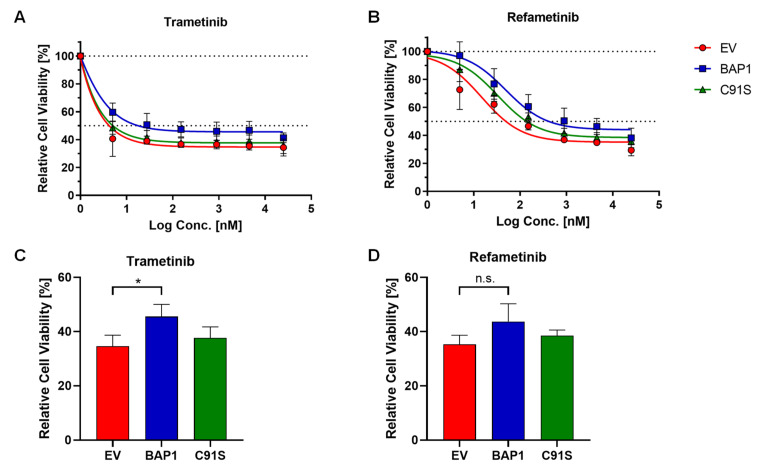
Reconstitution of wild-type *BAP1* does not sensitize UPMM2 cell line to MEK inhibition. UPMM2 cells were treated with trametinib (**A**) or refametinib (**B**) at different concentrations (from 5 nM to 25 µM) for 5 days and the minimum cell viability for each cell line after treatment was quantified (**C**,**D**). Cell viability is expressed as normalized values in percentage to DMSO controls. Summarized results of three independent experiments are shown. Data are average ± SD. *, *p* < 0.05. n.s., not significant.

Since *BAP1* mutations are occurring with high frequency in monosomy 3 UM tumors [24], we evaluated BAP1 protein expression in our six UM cell lines (Figure 2). By Western blot analysis we found that all D3 cell lines express BAP1 while no BAP1 was detected in any of the M3 cell lines, potentially implying the presence of *BAP1* mutations leading to protein loss in all M3 cell lines. These results are in agreement with other studies [33,34]. We therefore asked whether the resistance to MEK inhibitors (MEKi) could be driven by BAP1 loss. To address this, we tested whether an M3 cell line can be sensitized to MEKi by re-expressing wild-type *BAP1*. We first reconstituted wild-type *BAP1* or an inactive p.C91S mutant *BAP1* in the UPMM2 cell line and confirmed re-expression of BAP1 at the protein level by Western blot (Figure 3A). When constitutively expressing wild-type *BAP1*, cell proliferation significantly decreased (Figure 3B) and cells were less able to form colonies and proliferate when seeded at low density (Figure 3C), which is in line with the known role of *BAP1* as a tumor suppressor [28,29,30,35]. Reconstitution with the p.C91S mutant *BAP1* showed a similar cell proliferation as the empty vector (EV) control (Figure 3B) and even higher colony formation (Figure 3C). However, when treated with trametinib or refametinib, we did not observe a decrease in cell viability in wild-type *BAP1* reconstituted UPMM2 cells compared to the EV control (Figure 4). These findings suggest that BAP1 is not sufficient to influence the sensitivity to MEKi and perhaps other relevant interaction partners affected by monosomy 3 are necessary to drive the observed effect together with BAP1 loss.

### 2.3. Monosomy 3 Does Not Seem to Be Associated with Higher Activation of the ERK/MAPK Pathway

**Figure 5:**
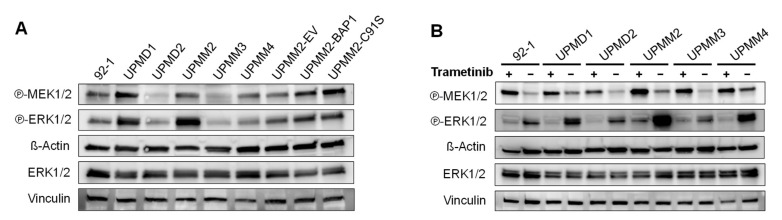
Expression levels of phosphorylated MEK and ERK in uveal melanoma cell lines in basal conditions and after treatment with trametinib. Western blot of phosphorylated MEK1/2 (Ser221), phosphorylated ERK1/2 (Thr202/Tyr204) and total ERK1/2 in basal conditions (**A**) or after treatment with 15 nM trametinib (or DMSO control) for 24 h (**B**). Total ERK1/2 and Vinculin were detected on an additional membrane using the same protein lysates. A representative result of two independent experiments is shown. Uncropped blots are displayed in Supplementary Figures S4 and S5.

To get further insight into the ERK/MAPK signaling pathway, we performed Western blotting for phosphorylated MEK and ERK for the D3 and M3 UM cell lines under basal conditions and upon treatment with 15 nM trametinib for 24 h (Figure 5). We observed a high variation in the levels of phosphorylated MEK1/2 at Ser221 and phosphorylated ERK1/2 at Thr202/Tyr204 among the UM cell lines without a clear distinction between the D3/M3 status (Figure 5A). In addition, reconstitution of UPMM2 with wild-type *BAP1* did not appear to change the phosphorylation of MEK or ERK.

Treatment with 15 nM trametinib for 24 h resulted in the inhibition of the ERK phosphorylation but an increase in the phosphorylation of MEK, which seemed slightly higher in the M3 cell lines compared to the D3 cell lines (Figure 5B). However, no striking differences were observed between the M3 and D3 cell lines regarding the phosphorylation status of MEK or ERK, suggesting that the resistance to MEKi might be explained by differences in other pathways.

### 2.4. UM Tumors from TCGA with BAP1 Mutations Show Monosomy 3 whereas Those with Wild-Type BAP1 Show Neutral DNA Copy Numbers

**Figure 6:**
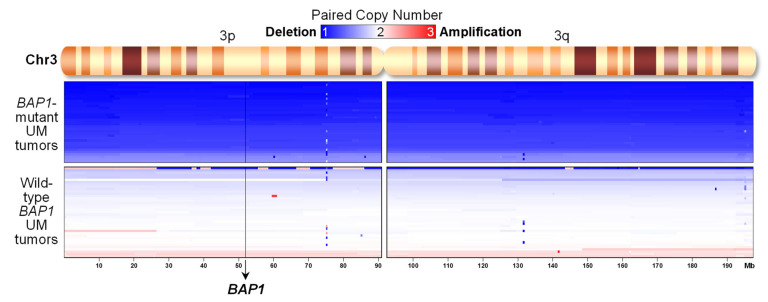
Uveal melanoma tumors from TCGA with *BAP1* mutations show deletion of chromosome 3, but not tumors with wild-type *BAP1*. Paired DNA copy numbers from UVM-TCGA tumors in chromosome 3 were sorted by their average segmented copy number (*n* = 74). The chromosomal position of the *BAP1* gene is indicated.

To get clinical significance of the differences in resistance to MEK inhibitors observed in UM cell lines, we analyzed 80 UM tumors from The Cancer Genome Atlas (TCGA) dataset [15]. We compared tumors with mutations in *BAP1* with those without *BAP1* mutations. An analysis of DNA copy number alterations (Figure 6) showed that all tumors with *BAP1* mutations displayed monosomy in chromosome 3 (an average DNA copy number of 1.2 ± 0.1) where *BAP1* gene is located, resulting in complete inactivation of BAP1. However, UM tumors with wild-type *BAP1* showed neutral copy numbers in chromosome 3 (an average DNA copy number of 1.9 ± 0.1, *p* = 6 × 10^−35^). Six tumors without mutations in *BAP1* but with deletions in chromosome 3 were considered unclear for the inactivation of BAP1 and discarded from further analyses. The remaining 74 tumors where then split into 35 samples with *BAP1* mutations and 39 without *BAP1* mutations.

### 2.5. UM Patients from TCGA with BAP1 Mutations Show Poorer Overall and Progression-Free Survival than Patients without Mutations in BAP1

**Figure 7:**
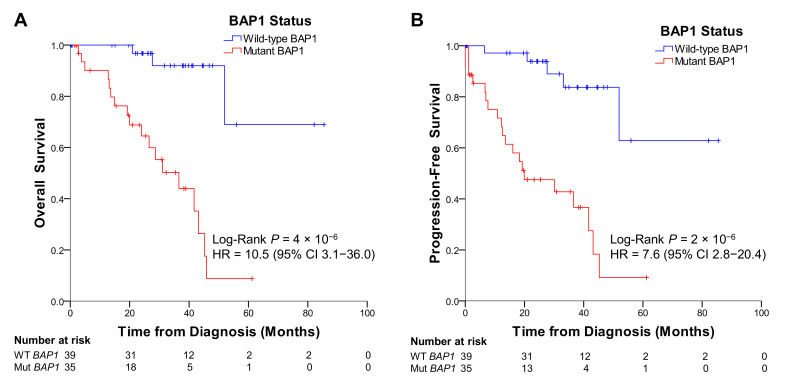
Uveal melanoma patients from TCGA with *BAP1* mutations display poor overall (**A**) and progression-free survival (**B**) compared to patients without mutations in *BAP1*. HR = hazard ratio.

UM patients from TCGA that had tumors with mutations in *BAP1* exhibited a much shorter overall survival (*p* = 4 × 10^−6^) and a higher incidence of metastases, indicated by a shorter progression-free survival (*p* = 2 × 10^−6^) than patients with wild-type *BAP1* (Figure 7). The hazard ratio for the mutations in *BAP1* was 10.5 (95% confidence interval [CI] 3.1–36.0) for overall survival and 7.6 (95% CI 2.8–20.4) for progression-free survival. This indicates the risk of dying or progressing for UM patients with tumors with *BAP1* mutations is about 10 and 8 times higher, respectively, than for patients with wild-type *BAP1*.

### 2.6. UM Patients with BAP1 Mutations Show a Completely Different Transcriptomics Program than Patients with Wild-Type BAP1

**Figure 8:**
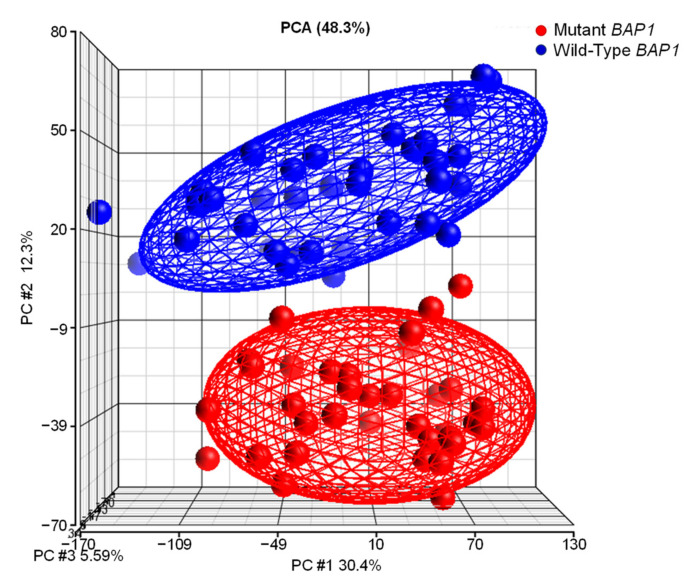
Transcriptome analysis of UVM-TCGA confidentially separates tumors with and without *BAP1* mutations. Gene expression profiling of uveal melanoma TCGA dataset (*n* = 74) followed by principal component analysis (PCA) using Partek Genomics Suite reveals distinct clustering of samples in two subgroups.

Next, we compared the gene expression profiling of tumors with and without *BAP1* mutations. Principal component analysis (PCA) of RNA-Seq data showed that they constitute completely different entities (Figure 8). Ellipsoids delineate the 95% confidence limit for each group and show that UM tumors with *BAP1* mutations cluster together and are clearly distinct from tumors with wild-type *BAP1*.

**Figure 9:**
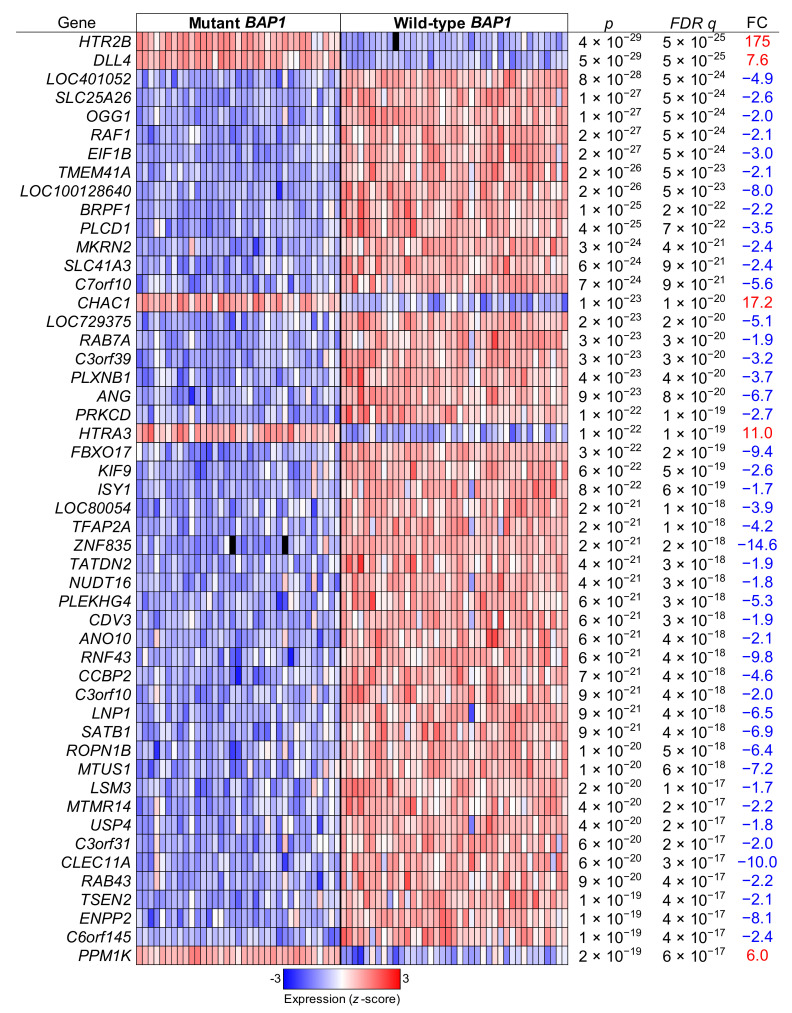
Top differentially regulated genes in *BAP1*-mutant versus *BAP1*-wild type UM tumor samples. Uveal melanoma TCGA data (*n* = 74) were analyzed and grouped in mutant *BAP1* and wild-type *BAP1* tumor samples. The top significantly differentially regulated genes after a false discovery rate (FDR) corrected *p* value are shown. FC, fold change.

We then compared the gene expression of tumors with *BAP1* mutations to those with wild-type *BAP1* using *t* tests and correcting the *p* values with the Benjamini and Hochberg false discovery rate (FDR) correction method. That lead to the identification of 7130 differentially expressed genes after an FDR *q* < 0.05 (3988 upregulated genes and 3142 downregulated genes). The top 50 differentially expressed genes are displayed in Figure 9.

To gain better insight into the gene expression profile characteristics of UM tumors with *BAP1* mutations, we performed gene expression network analysis with Qiagen Ingenuity Pathway Analysis (IPA) software using a stringent list of differentially expressed genes (an FDR *q* < 10^−10^ and a fold change > 2 in both directions). Surprisingly, the most significant network showed an indirect link between BAP1 and ERK (Supplementary Figure S6), which supports our observations of resistance to MEKi in UM cell lines with BAP1 (Figure 1).

To find the overrepresented pathways caused by mutations in *BAP1*, we selected genes that met a relaxed criterion (an FDR *q* < 0.001 and a fold change > 1.5 in both directions) and a stringent criterion (an FDR *q* < 10^−10^ and a fold change > 2 in both directions). We performed IPA for both gene lists and identified their overrepresented canonical pathways. The top 35 enriched canonical pathways significant in both gene lists are displayed in Table 1. Of note, the ERK/MAPK signaling pathway is the 31st most significant pathway (*p* = 0.001) (Supplementary Figures S7 and S8), indicating that this pathway might not be the most critical to explain the differences for *BAP1*-mutant versus wild-type *BAP1* tumors.

### 2.7. The eIF2 Signaling Is the Most Overrepresented Pathway in BAP1-Mutant UM Tumors

**Figure 10:**
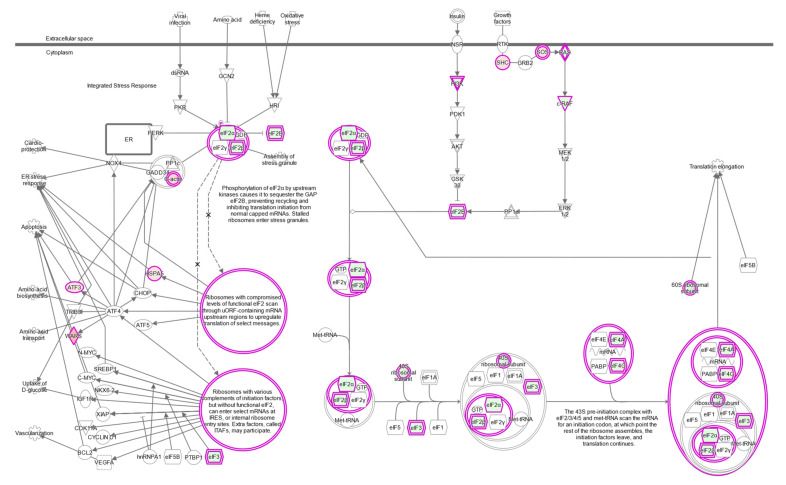
eIF2 signaling pathway. Ingenuity pathway analysis shows upregulated genes in shades of red and downregulated genes in shades of green in *BAP1*-mutated UVM-TCGA tumors.

The eukaryotic initiation factor 2 (eIF2) signaling pathway is the most significant pathway identified by IPA comparing wild-type to mutant *BAP1* UM tumors (*p* = 4 × 10^−10^) (Table 1, Figure 10). Similar significant pathways involved in the regulation of protein translation that share some key genes are the regulation of eIF4 and p70S6K signaling (*p* = 4 × 10^−7^) and mTOR signaling (*p* = 7 × 10^−4^) (Table 1).

**Figure 11:**
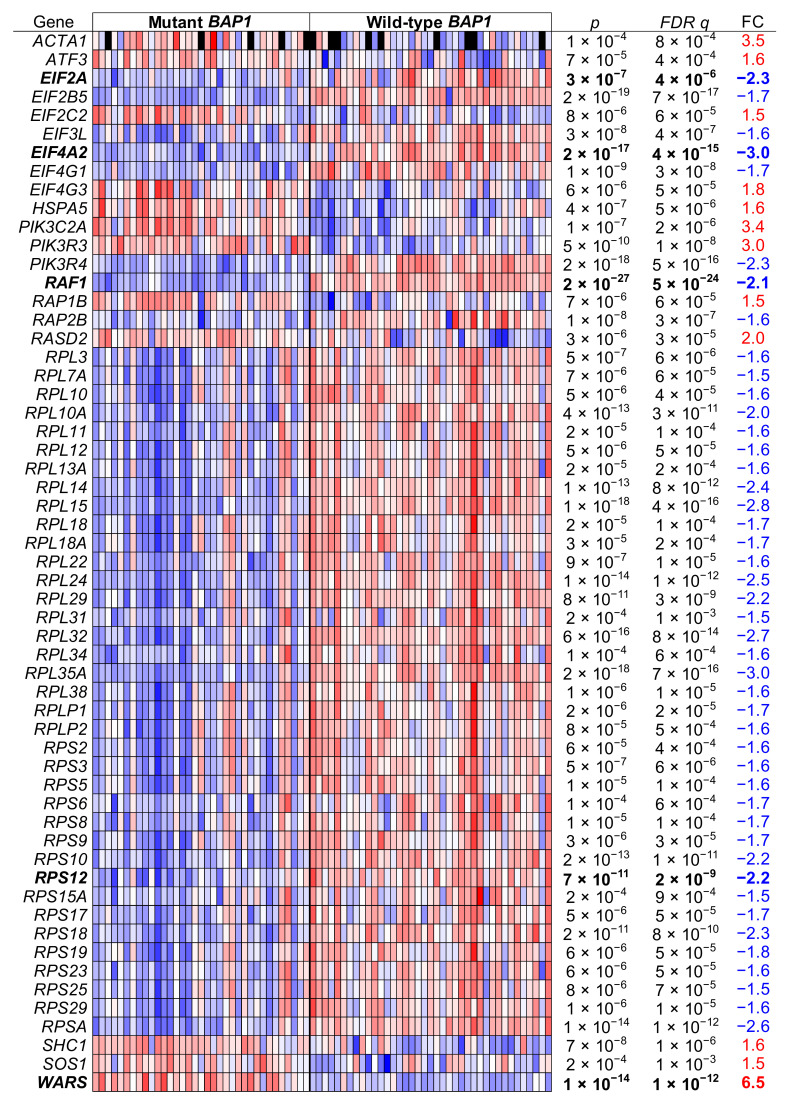
eIF2 signaling pathway genes in *BAP1*-mutant and *BAP1*-wild type UM tumor samples. Uveal melanoma TCGA data (*n* = 74) were analyzed and grouped in *BAP1*-mutant and *BAP1*-wild type tumor samples. The genes of the eIF2 signaling pathway that were differentially expressed after a false discovery rate (FDR) corrected *p* value below 0.001 and a fold change (FC) higher than 1.5 are shown. Genes further assayed by qPCR are in bold.

A closer look at the genes differentially expressed revealed that UM tumors with *BAP1* mutations displayed a downregulation in *EIF2A* and in the expression of ribosomal protein genes for the large and small subunits in a coordinated manner (Figure 11).

**Figure 12:**
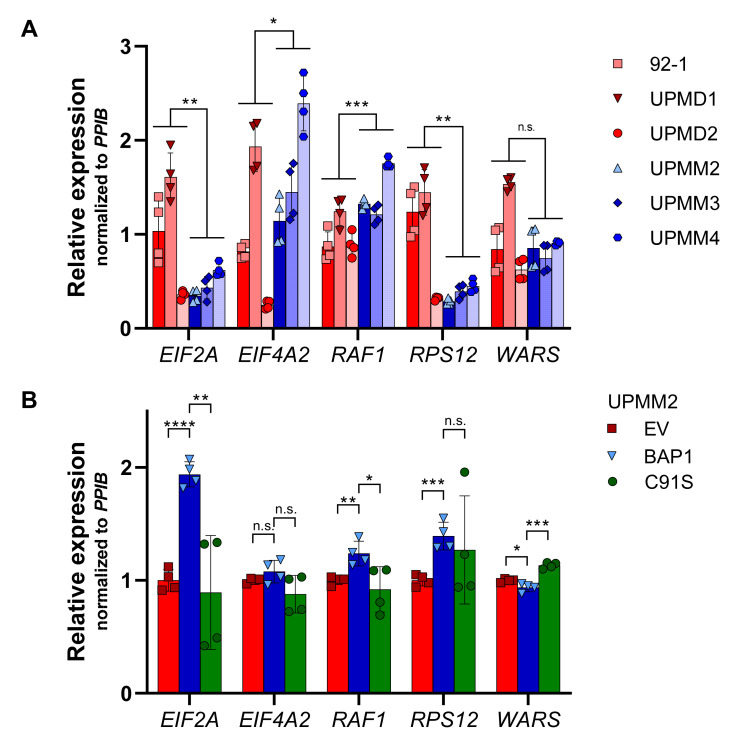
Gene expression of eIF2 pathway-related genes in UM cell lines. Expression of five eIF2 pathway-related genes was assessed by qPCR in UM cell lines (**A**) and *BAP1*-reconstituted UPMM2 cell lines (**B**). Gene expression was normalized to *PPIB* levels relative to the average expression levels in D3 cell lines or the empty vector (EV) control. Summarized results of two biological replicates are shown (cells in B were from the same passage). Data are average ± SD. *, *p* < 0.05. **, *p* < 0.01. ***, *p* < 0.001. ****, *p* < 0.0001. n.s., not significant.

We assayed by qPCR some of the significantly differentially regulated genes of the eIF2 signaling pathway listed in Figure 11 in the UM cell lines with D3 and M3. We validated the expression of *EIF2A* and ribosomal protein S12 (*RPS12*) by qPCR, which showed lower gene expression levels in the M3 cell lines than in the D3 cell lines (*p* < 0.01, Figure 12A), in agreement with the RNA-Seq results in *BAP1*-mutant UM tumors (Figure 11). However, gene expression for *EIF4A2* and *RAF1* in M3 cell lines was higher than in D3 cell lines (*p* < 0.05 and *p* < 0.001, respectively, Figure 12A), which is the opposite as to what was observed in UVM-TCGA tumors (Figure 11), while tryptophanyl-tRNA synthetase (*WARS*) showed no significant differences (Figure 12A).

Reconstitution of wild-type but not mutant *BAP1* in the BAP1-deficient UPMM2 cell line increased the expression of *EIF2A* two-fold (*p* < 0.0001 and *p* < 0.01, respectively, Figure 12B). This phenomenon was also observed to a lower extent for *RAF1* and *RPS12* and the contrary for *WARS* (Figure 12B), in agreement with what would be expected based on expression in TCGA tumors (Figure 11).

## 3. Discussion

Metastatic UM is a rare but fatal disease that is elusive from current therapies, including chemotherapy and immune checkpoint inhibitors [7]. In order to find potential new therapeutic approaches for the treatment of UM, we evaluated the response of six UM cell lines to a variety of chemotherapeutic and targeted compounds. Here, we found cell lines with M3 to be significantly more resistant to treatment with MEK inhibitors compared to cell lines with D3. MEK inhibitors have been proven to be effective in cancers with constitutive activation of the MAPK/ERK pathway and have been investigated in UM, which displays *GNAQ/GNA11*-mutations in >90% of cases [17,18]. However, in clinical trials, MEK inhibitor therapy did not add any benefits to conventional palliative chemotherapy in UM [19,20]. Since metastatic disease mostly arises from M3 tumors, our findings of M3 tumors being resistant to MEK inhibition is in line with the failure of MEK inhibitors in clinical studies [21,22]. Understanding the mechanisms behind the resistance of aggressive M3 tumors to this inhibitor class can build a path for finding vulnerabilities in UM.

As *BAP1* is frequently mutated in UM and associated with the more aggressive M3 phenotype [24,32], we performed experiments to evaluate the potential role of *BAP1* in resistance to MEK inhibition. We identified loss of BAP1 protein expression in all M3 cell lines, confirming the proposed association of *BAP1* mutations and M3. When re-expressing wild-type *BAP1* in M3 cell line UPMM2, we detected a substantial decrease in proliferation rate and reduced ability to form colonies, which supports the discussed role of *BAP1* as a tumor suppressor (reviewed in [35]). Similar effects were observed in a study by Chen and colleagues, where reconstitution of wild-type *BAP1* in a *BAP1*-mutated intrahepatic cholangiocarcinoma (ICC) cell line led to decreased cell proliferation, cell cycle progression and invasion, while knockdown of *BAP1* in a *BAP1*-wild type ICC cell line showed the opposite effect [36]. Interestingly, it has been shown by Matatall and colleagues that stable or transient knockdown of *BAP1* in *BAP1*-wild type UM cell lines resulted in no changes or even in a decrease of in vitro tumorigenicity, respectively, defined by proliferation rate, migration and invasion ability [31]. Due to these controversial findings, it can be assumed that the adequacy of common in vitro models for drawing conclusions on the exact role of *BAP1* as a tumor suppressor is limited, and that effects of BAP1 loss on tumor aggressiveness are highly plastic and probably regulated by a variety of other factors that need to be elucidated. Thus, reconstitution of the BAP1-deficient UPMM2 cell line with wild-type *BAP1* was unable to sensitize cells to MEK inhibitors, suggesting that BAP1 expression is not sufficient to confer sensitivity in the context of chromosome 3 loss.

To further understand the implications of the ERK/MAPK pathway in the mechanism of resistance of the M3 cell lines to MEK inhibitors, we compared the phosphorylation state of MEK and ERK for the D3 and M3 cell lines at basal levels and upon treatment with trametinib. However, no significant differences were observed in our experimental conditions, suggesting that other pathways might be implicated in the mechanism of resistance to MEKi in UM cell lines.

To evaluate other potential pathways implicated in the mechanism of resistance to MEKi, we analyzed clinical and genomic data from TCGA of UM patients. Our analysis of UVM-TCGA data confirms that the occurrence of *BAP1*-mutations is highly associated with M3, as has previously been shown, supporting the hypothesis of M3 unmasking recessive *BAP1*-mutations in UM [24]. The original UVM-TCGA analysis reported by Robertson and colleagues [15] classified UM in four groups, two with D3 and high incidence of mutations in *EIF1AX* and *SRSF2/SF3B1*, which showed good prognosis, and two groups with M3 and high incidence of *BAP1* mutations, whose patients displayed poor prognosis. However, there was no direct comparison of tumors with *BAP1* mutations with those with wild-type *BAP1*. Here, we removed samples with unclear *BAP1* mutation/inactivation and compared UM tumors based on their *BAP1* mutational status. We show that UM tumors can be classified in two distinct groups according to their *BAP1* mutational status, with differential gene expression profiles and *BAP1* mutations associated with poor prognosis.

Due to the strong association of *BAP1* mutations with M3 in UM, differences in gene expression and prognosis could be a result of differential chromosome 3 status independent of *BAP1.* The options for investigating the exact role of *BAP1* independent of M3 as a driver of tumor aggressiveness in UM using genomic and transcriptomic patient data are limited because of the lack of data from M3 tumors without *BAP1* mutations. This tight bond of M3 and mutations in *BAP1* is by itself a strong indicator of the presence of both factors being necessary for a metastasizing, aggressive UM phenotype. It has been shown previously that loss of BAP1 expression can be utilized as a prognostic marker in RCC and breast cancer [30,37]. Shah and colleagues investigated the prognostic value of *BAP1* mutations and loss of BAP1 expression in UM patient tissue and showed a significant correlation between BAP1 loss and poor survival [32], whose findings were later confirmed by others [38,39,40] and are supported by our results when analyzing the UVM-TCGA dataset. Despite *BAP1* being seemingly necessary but not sufficient to confer sensitivity to MEK inhibition in our setting, we detected an indirect link between BAP1 and ERK in the most significant network based on gene expression network analysis of UVM-TCGA data when analyzing *BAP1* mutant compared to wild-type *BAP1* (representative for M3 compared to D3) tumors. In a mouse model of mesothelioma, conditional knockout of *Bap1* increased phosphorylation of ERK and accelerated tumor development, especially in the presence of accompanying *Nf2* and *Cdkn2ab* deletions [41], emphasizing the relevance of higher aggressiveness observed by inactivating additional tumor suppressors.

In general, all our UM cell lines display low proliferation rates, and the M3 cell lines we used have shown significantly lower replicative potential compared to the D3 cell lines. While lower proliferation rates in M3 compared to D3 cell lines were observed, the differences were not significant [42]. Similarly, we observed a significant increase in resistance to trametinib after reconstitution of wild-type *BAP1* in UPMM2, when reconstitution at the same time caused a decrease in proliferation. Since we could not detect the observed resistance pattern when cells were treated with other compounds, we hypothesize that, if at all, replication/proliferation differences selectively play a role in altering sensitivity to MEK inhibitors, perhaps by cell cycle-dependent differences in MEK/ERK activation, which has been reported for myeloid leukemia cell lines [43]. Further experiments to address the relevance of differential replicative potential and proliferation rates on MEK inhibitors sensitivity in UM are required. In addition, even if not affecting sensitivity to MEK inhibition on the level of cell viability, possible differences in the expression of phosphorylated MEK and ERK upon MEK inhibition in *BAP1*-reconstituted UM cells will need to be addressed in future work.

Besides BAP1 loss itself as a possible modulator of sensitivity to MEK inhibition in M3-UM, we identified noteworthy pathway alterations. The most significantly overrepresented pathway in *BAP1*-mutant (M3) tumors with potential relevance in affecting sensitivity to MEK inhibition is *eIF2 Signaling*. eIF2 signaling is critical for the integrated stress response (ISR), which can be induced by different sources of cellular stress. One important source of cellular stress is endoplasmic reticulum (ER) stress, characterized by an accumulation of unfolded proteins in the ER and an activation of the unfolded protein response (UPR) pathway. One major effector activated in the UPR is double-stranded RNA-activated protein kinase (PKR)-like endoplasmic reticulum kinase (PERK). PERK, also known as eIF2AK3, is activated by stress-induced molecular chaperones, e.g., GRP78, and in turn phosphorylates eukaryotic initiation factor 2α (eIF2α). Active peIF2α mediates translational attenuation and expression of a variety of ISR-related genes, resulting in two possible outcomes that depend on the intensity and duration of ER stress: survival or apoptosis (reviewed in [44,45,46]). It has been shown that MEK inhibition triggers ER stress resulting in UPR pathway activation, defined by an increased expression of GRP78 and induction of PERK/peIF2α signaling [47,48]. Differential expression of key players in this pathway, as observed in our study in M3-UM tumors, could affect MEK inhibitor-induced ISR and sensitivity to MEK inhibition. To address this possibility, in vitro and in vivo experiments investigating differential ISR regulation in M3-UM cells compared to D3-UM cells upon MEK inhibition are necessary.

Most cancer cells support cell proliferation and growth by deregulating oncogenic pathways implicated in mRNA translation. Thus, considering that *BAP1*-mutant UM tumors are more aggressive than those with wild-type *BAP1*, it would be expected that they have a higher capacity of synthesizing proteins. Conversely, we observed a systematic downregulation of the ribosomal protein genes along with downregulation of *EIF2A*, which we validated by qPCR, and might result in a coordinated decrease of ribosome biogenesis in *BAP1*-mutant tumors. In fact, under certain stress conditions in the tumor microenvironment such as hypoxia, nutrient deprivation, and oxidative and genotoxic stress, inhibition of eIF2 can trigger coordinated decreases in protein biosynthesis to preserve energy and prevent the accumulation of damaged and misfolded proteins. This in turn allows for the selective translation of mRNAs critical in the adaptive response to stress (reviewed in [49]). Thus, our data suggests that *BAP1*-mutant UM tumors and M3 cell lines might be more resistant to MEK inhibitors because they are able to downregulate eIF2 to diminish global ribosome biogenesis while simultaneously inducing genes essential to the adaptive stress response.

Taken together, our study reveals decreased sensitivity to MEK inhibition in M3-UM cell lines that was not restored by reconstitution of wild-type *BAP1*. Despite seemingly not having a direct effect on susceptibility to MEK inhibition, we show that UM tumors can be classified in two tumor subgroups with different clinical outcomes based on their *BAP1* mutation status by analyzing UVM-TCGA data. *BAP1*-mutant tumors display differential expression of key genes involved in canonical pathways that could play critical roles in modulating the response to MEK inhibition, such as downregulation of the eIF2 signaling to decrease ribosome biogenesis. Since M3-UM tumors define a highly aggressive cancer entity resistant to common therapies, understanding the implications of differentially regulated pathways and resulting resistance mechanisms is indispensable for identifying new ways of targeting this lethal disease. Further investigation of the possible relevance of the aforementioned signaling pathways in therapy resistance is highly needed.

## 4. Materials and Methods

### 4.1. Cell Lines

Uveal melanoma cell lines UPMD1, UPMD2, UPMM2, UPMM3 and UPMM4 were kindly provided as early passages by the Department of Human Genetics, University Hospital Essen (Dr. M. Zeschnigk) and were derived from untreated primary uveal melanomas and characterized regarding their chromosome 3 status [42]. Primary-derived cell lines were cultured in Ham’s F-12 Nutrient Mix (Thermo Fisher Scientific, Waltham, MA, USA, 31765068) supplemented with 10% (*v*/*v*) fetal bovine serum (FBS, Life Technologies, 10500064) and 1% (*v*/*v*) Penicillin-Streptomycin (Thermo Fisher Scientific, 15140122). Cell line 92-1 was cultured in RPMI 1640 medium (Thermo Fisher Scientific, 61870044) supplemented with 10% FBS and 1% Penicillin-Streptomycin. All cell lines were routinely tested for mycoplasma by PCR.

### 4.2. BAP1 Reconstitution

For stable expression of wild-type *BAP1* or inactive mutant p.C91S *BAP1* in the *BAP1*-deficient M3 cell line UPMM2, retroviruses were generated in HEK-293GP cells cultured in DMEM medium (Thermo Fisher Scientific, 41965062) supplemented with 10% FBS and 1% Penicillin-Streptomycin. HEK-293GP cells were transfected with pVSV-G envelope plasmid [50] (Addgene, Watertown, MA, USA, 8454) and the constructs pBABE-hygro [51] (Addgene, 1765), pBABE-hygro-BAP1-HA [52] (Addgene, 154020) or pBABE-hygro-BAP1-C91S-HA [52] (Addgene, 154021), respectively, using TransIT-LT1 Transfection Reagent (Mirus, Madison, WI, USA, 731-0029) according to the manufacturer’s protocol. Virus-containing medium was collected and filtered through a 0.45 µm syringe filter (Corning, Wiesbaden, Germany, 431220). Culture medium of UPMM2 cells was replaced with filtered virus-containing medium for transduction and 48 h after transduction, cells were selected with 100 µg/mL hygromycin B (Santa Cruz, Dallas, TX, USA, sc-29067) for 1 week.

### 4.3. Drug Treatment

Trametinib (Selleck Chemicals, Houston, TX, USA, S2673) and Refametinib (Selleck Chemicals, S1089) were dissolved to 10 mM in DMSO and printed at nine concentrations from 5 nM to 25 µM in triplicates in white-bottom sterile polystyrene 384-well plates (Corning, Wiesbaden, Germany, 3570) using a Tecan D300e Digital Dispenser (Tecan, Männedorf, Switzerland/HP Inc., Palo Alto, CA, USA) and the corresponding software D300e Control (v3.1.3, Tecan). DMSO was printed as a negative control. Pre-printed plates were sealed and stored at −80 °C until used. Prepared assay plates were equilibrated at room temperature and 500 cells per well were seeded in 30 µL culture medium with a Multidrop Combi dispenser (Thermo Fisher Scientific). Assay plates were incubated at 37 °C and 5% CO_2_ for 5 days (120 h).

### 4.4. Cell Viability Assay

CellTiter Glo reagent (Promega, Madison, WI, USA, G7573) was prepared according to the manufacturer’s protocol. Assay plates were equilibrated at room temperature for 30 min and 30 µL of a 1:4 dilution of CellTiter Glo reagent in Dulbecco’s phosphate-buffered saline (DPBS, Thermo Fisher Scientific, 14190169) were added to each well. Plates were shaken for 2 min and incubated for 8 min at room temperature protected from light. Luminescence was measured at 500 milliseconds using a Tecan Spark 10 M microplate reader (Tecan) and the corresponding software Spark Control (v1.2, Tecan). Cell viability was normalized to non-treated DMSO controls and data was analyzed in Microsoft Excel (v16.0.13801.20288, Microsoft, Redmond, WA, USA) and GraphPad Prism 9 (v9.0.0, GraphPad, San Diego, CA, USA).

### 4.5. Proliferation Assay

Five hundred cells of *BAP1*-reconstituted UPMM2 cells per well were seeded in 30 µL culture medium in triplicates in white-bottom sterile polystyrene 384-well plates (Corning, Wiesbaden, Germany, 3570) in seven replicate plates per experiment and incubated at 37 °C and 5% CO_2_. Every 24 h, cell viability was assessed via CellTiter Glo viability assay (see Section 4.4) as a surrogate for proliferation for 7 days and values were normalized to day 1. Data was analyzed in Microsoft Excel (v16.0.13801.20288) and GraphPad Prism 9 (v9.0.0).

### 4.6. Colony Formation Assay

*BAP1*-reconstituted UPMM2 cells (3000) per well were seeded in 2 mL culture medium in duplicates in 6-well cell culture plates. Even distribution of single cells was ensured via microscopy. Assay plates were incubated for 5 weeks at 37 °C and 5% CO_2_. Culture medium was refreshed every 3–5 days. After 5 weeks, culture medium was aspirated, and cells were washed 1× with cold DPBS (Thermo Fisher Scientific, 14190169) and placed on ice. For fixation, 1 mL of cold methanol (J.T. Baker, Phillipsburg, NJ, USA, 8045) was added per well and incubated for 10 min on ice under gentle agitation. Methanol was removed and 1 mL of 0.1% crystal violet (Sigma-Aldrich, St. Louis, MO, USA, C0775) in 25% (*v*/*v*) methanol was added and incubated for 30 min at room temperature under mild agitation. Staining solution was removed, wells were washed thoroughly with tap water and plates were air-dried upside-down on tissue paper. Plates were scanned using an Epson Perfection V850 Pro Scanner (Epson, Suwa, Japan) and the corresponding software Epson Scan (v.3.9.3.4, Epson). Confluency was quantified as previously described using the ImageJ-Plugin “ColonyArea” [53]. Data was analyzed in Microsoft Excel (v16.0.13801.20288) and GraphPad Prism 9 (v9.0.0).

### 4.7. Western Blot

Western blot experiments were performed as described previously [52]. In brief, cells were harvested in Protein Lysis buffer [54] containing protease (Thermo Fisher Scientific, 11834101) and phosphatase inhibitors (Thermo Fisher Scientific, 11814101). About 8–10 μg of protein was resolved under denaturing conditions (SDS-PAGE) and transferred to a 0.2 μm nitrocellulose membrane. Membranes were blocked in 5% bovine serum albumin (Sigma-Aldrich, A7906) in TBS-T (500 mM Tris, 1.5 M NaCl, 0.1% Tween-20, pH 7.6) and incubated in primary monoclonal rabbit anti-phosphorylated MEK1/2 (Ser221) antibody (Cell Signaling, Danvers, MA, USA, 2338S), monoclonal rabbit anti-phosphorylated ERK1/2 (Thr202/Tyr204) antibody (Cell Signaling, 4376S), monoclonal rabbit anti-ERK1/2 antibody (Cell Signaling, 4695S) and monoclonal mouse anti-BAP1 (C-4) antibody (Santa Cruz, sc-28383) diluted 1:1000 in 5% BSA/TBS-T containing 0.05% sodium azide overnight at 4 °C. Membranes were washed in TBS-T and incubated in HRP-conjugated polyclonal goat anti-mouse IgG secondary antibody (Jackson ImmunoResearch, Ely, UK, 115-035-003) diluted 1:2000 in 5% BSA/TBS-T for 1 h at room temperature prior to visualization in a ChemiDoc MP Image System (Bio-Rad, Hercules, CA, USA). As a loading control, HRP-conjugated monoclonal mouse anti-ß-Actin (C-4) antibody (Santa Cruz, sc-47778-HRP) and monoclonal rabbit anti-vinculin antibody (Cell Signaling, 13901S) were used.

### 4.8. The Cancer Genome Atlas (TCGA) Data Acquisition

RNA-Sequencing (RNA-Seq) and segmented DNA copy numbers (GRCh37/hg19) data from 80 uveal melanoma (UVM) patients from The Cancer Genome Atlas (TCGA) were downloaded from the Genomic Data Commons (GDC) data portal on 19 December 2020 (https://portal.gdc.cancer.gov). *BAP1* mutation status was obtained from Robertson and colleagues [15]. Six tumors without mutations in *BAP1* but with monosomy in chromosome 3 were considered uncertain for BAP1 inactivation and were discarded from further analyses.

### 4.9. UM Patient Survival Analysis

Metastasis and overall survival data were obtained from Robertson et al. (2017) [15] and analyzed as previously described [55]. Progression-free survival considered the time to metastasis or death of the patient. Kaplan-Meier survival curves, log-rank tests and Cox regression models were calculated with IBM SPSS Statistics 25.0. The hazard ratio (HR) and the 95% confidence intervals (CI) of *BAP1* mutations were estimated using univariate Cox regression models.

### 4.10. DNA Copy Number Analyses

Segmented DNA copy numbers (GRCh37/hg19) were interrogated every 0.5 Mb in chromosome 3 to display the paired copy number for each UVM-TCGA tumor, which were sorted by their averaged copy number in chromosome 3.

### 4.11. Gene Expression Analyses

Gene expression levels were estimated by the RNA-Seq Expectation-Maximization (RSEM) normalization method and log2 transformed. Gene expression data were analyzed as previously reported [56]. Briefly, unpaired *t* tests were performed between tumors with mutations in *BAP1* and those without mutations in *BAP1* considering the group variances. All calculated *p* values were corrected using the Benjamini and Hochberg false discovery rate (FDR) method to discard false positives by the fact of performing multiple tests. Principal Component Analysis (PCA) was computed with Partek Genomics Suite (v9.0, Partek, St. Louis, MO, USA) using all transcripts expressed in the 74 UM tumors (13,164). Significant genes with an FDR *q* < 10^−3^ and a fold change (FC) > 1.5, as well as genes with FDR *q* < 10^−10^ and FC > 2 were further analyzed with Ingenuity Pathway Analysis (IPA) software Spring Release (March 2021), v62089861 (Qiagen, Hilden, Germany) software to identify overrepresented canonical pathways for *BAP1* mutations.

### 4.12. Quantitative Reverse-Transcribed PCR (qRT-PCR)

RNA was extracted using the AllPrep DNA/RNA Kit (Qiagen, 80204) according to the manufacturer’s protocol. RNA was quantified using a Nanodrop 2000c spectrophotometer (Thermo Fisher Scientific) and 1 µg or 2 µg of RNA were reverse transcribed to cDNA using the High Capacity cDNA Reverse Transcription Kit (Thermo Fisher Scientific, 4368814) according to the manufacturer’s instructions. Conventional PCR was performed with cDNA samples obtained from 2 µg RNA using the Hot Star Taq Plus Master Mix Kit (Qiagen, 203643) according to the manufacturer’s protocol. PCR products were evaluated on a 2% agarose gel to assess primer specificity and gel-purified using the QIAquick Gel Extraction Kit (Qiagen, 28706) and quantified. Serial dilutions of each amplicon were generated to serve as standards of known concentrations during quantitative reverse-transcribed PCR (qRT-PCR). qRT-PCR was performed on diluted cDNA samples or standards of known concentrations using LightCycler 480 SYBR Green I Master Mix (Roche, Basel, Switzerland, 4887352001) on a LightCycler 480 (Roche) with the following conditions: 95 °C for 5 min and 45 cycles of 95 °C for 10 s, 60 °C for 10 s and 72 °C for 15 s. Melting curves were generated for each sample. Gene expression was analyzed in Microsoft Excel (v16.0.13801.20288) and GraphPad Prism 9 (v9.0.0) by generating standard curves from known amplicon dilutions and interpolating sample Cp values for absolute quantification. Data was normalized to the housekeeping gene peptidylprolyl isomerase B (*PPIB*), also known as cyclophilin B, and plotted as relative values. Primer sequences are shown in Supplementary Table S1.

## Figures and Tables

**Table 1:** Top canonical pathways for genes differentially expressed between uveal melanoma tumors from TCGA with and without *BAP1* mutations when using both *t* tests corrected by a false discovery rate (FDR) *q* < 10^−3^ with fold change (FC) > 1.5 and an FDR *q* < 10^−3^ with FC > 2. Important pathways are in bold.

Canonical Pathways	FDR *q* < 10^−3^, FC > 1.5		FDR *q* < 10^−10^, FC > 2	
	*p*	Ratio	*p*	Ratio
**eIF2 Signaling**	4 × 10^−10^	0.251	5 × 10^−6^	0.053
Axonal guidance signaling	6 × 10^−9^	0.192	0.006	0.024
Cardiac hypertrophy signaling (enhanced)	10^−7^	0.183	3 × 10^−4^	0.030
**Regulation of eIF4 and p70S6K signaling**	4 × 10^−7^	0.241	0.009	0.035
Synaptogenesis signaling pathway	10^−6^	0.197	0.049	0.022
Phospholipase C signaling	3 × 10^−6^	0.201	0.008	0.029
GP6 signaling pathway	4 × 10^−6^	0.246	0.046	0.031
Gap junction signaling	9 × 10^−6^	0.209	0.006	0.033
T cell receptor signaling	10^−5^	0.252	0.028	0.036
Thrombin signaling	10^−5^	0.206	8 × 10^−5^	0.047
Tec kinase signaling	2 × 10^−5^	0.216	0.010	0.034
CREB signaling in neurons	2 × 10^−5^	0.161	0.004	0.023
Role of NFAT in cardiac hypertrophy	3 × 10^−5^	0.199	2 × 10^−5^	0.050
Phagosome formation	5 × 10^−5^	0.226	0.003	0.045
Protein kinase A signaling	5 × 10^−5^	0.170	0.011	0.024
Cardiac hypertrophy signaling	7 × 10^−5^	0.189	0.004	0.032
Natural killer cell signaling	8 × 10^−5^	0.198	0.005	0.035
Glioblastoma multiforme signaling	9 × 10^−5^	0.206	0.009	0.035
HIF1α signaling	2 × 10^−4^	0.190	0.023	0.028
Hepatic fibrosis signaling pathway	2 × 10^−4^	0.165	0.020	0.023
Sphingosine-1-phosphate signaling	4 × 10^−4^	0.215	0.037	0.033
Adrenomedullin signaling pathway	5 × 10^−4^	0.185	0.006	0.034
Breast cancer regulation by stathmin1	5 × 10^−4^	0.150	0.001	0.025
Glioma signaling	6 × 10^−4^	0.212	0.008	0.042
Molecular mechanisms of cancer	6 × 10^−4^	0.159	0.026	0.022
**mTOR Signaling**	7 × 10^−4^	0.181	0.007	0.032
Sperm motility	7 × 10^−4^	0.176	0.012	0.029
Endothelin-1 signaling	7 × 10^−4^	0.182	3 × 10^−4^	0.044
cAMP-mediated signaling	8 × 10^−4^	0.176	0.035	0.026
14-3-3-mediated signaling	10^−3^	0.202	0.002	0.047
**ERK/MAPK signaling**	0.001	0.180	0.020	0.029
HGF signaling	0.001	0.203	0.002	0.049
Xenobiotic metabolism signaling	0.001	0.166	9 × 10^−4^	0.035
Production of nitric oxide and ROS in macrophages	0.001	0.179	0.016	0.031
CXCR4 signaling	0.002	0.183	0.002	0.040

## Supplementary Materials

The following are available online at https://www.mdpi.com/article/10.3390/ijms22136727/s1, Figure S1: MEKi resistance pattern of M3 cell lines is not recapitulated in experiments with other compounds, Figures S2–S5: Uncropped Western Blots, Figure S6: BAP1 regulates ERK indirectly, Figure S7: ERK/MAPK Signaling, Figure S8: ERK/MAPK signaling pathway genes in BAP1-mutant and BAP1-wild type UM tumor samples, Table S1: List of primers.

## Abbreviations

BAP1	BRCA1-associated protein 1
BRCA	Breast cancer gene
BSA	Bovine serum albumin
CI	Confidence intervals
D3	Disomy 3
EGFR	Endothelial growth factor receptor
eIF2α	Eukaryotic initiation factor 2α
ER	Endoplasmic reticulum
FBS	Fetal bovine serum
FC	Fold change
FDR	False discovery rate
GDC	Genomic Data Commons
ICC	Intrahepatic cholangiocarcinoma
IPA	Ingenuity pathway analysis
ISR	Integrated stress response
M3	Monosomy 3
MAPK	Mitogen-activated protein kinase
MEKi	MEK inhibitor(s)
PARP	Poly-ADP (adenosine diphosphate)-ribose polymerase
PCA	Principal component analysis
PERK	Double-stranded RNA-activated protein kinase (PKR)-like endoplasmic reticulum kinase
PPIB	Peptidylprolyl isomerase B
q(RT)-PCR	Quantitative (reverse-transcribed) PCR
RCC	Renal cell carcinoma
RPS12	Ribosomal protein S12
TCGA	The Cancer Genome Atlas
UM	Uveal melanoma
UPR	Unfolded protein response
