# Supplementary material for: Monosomy 3 Is Linked to Resistance to MEK Inhibitors in Uveal Melanoma
Source: Int J Mol Sci. 2021 Jun 23;22(13):6727. doi: 10.3390/ijms22136727 (PMC8269285; doi:10.3390/ijms22136727)
Supplement: Supplementary file 1 [file ijms-22-06727-s001.zip › ijms-1225725-supplementary.pdf]

Supplementary Materials

Monosomy 3 is linked to resistance to MEK inhibitors in uveal melanoma

Svenja Mergener[1,2,3], Jens T. Siveke[2,3] and Samuel Peña-Llopis[1,2,3,*]

[1]Translational Genomics in Solid Tumors, German Cancer Consortium (DKTK) and German Cancer Research Center (DKFZ) at the University Hospital Essen, Hufelandstrasse 55, D-45147 Essen, Germany.

[2]Division of Solid Tumor Translational Oncology, German Cancer Consortium (DKTK) and German Cancer Research Center (DKFZ), Heidelberg, Germany.

[3]Bridge Institute of Experimental Tumor Therapy, West German Cancer Center, University Hospital Essen, Essen, Germany.

*Correspondence: Samuel.Pena-Llopis@dkfz.de

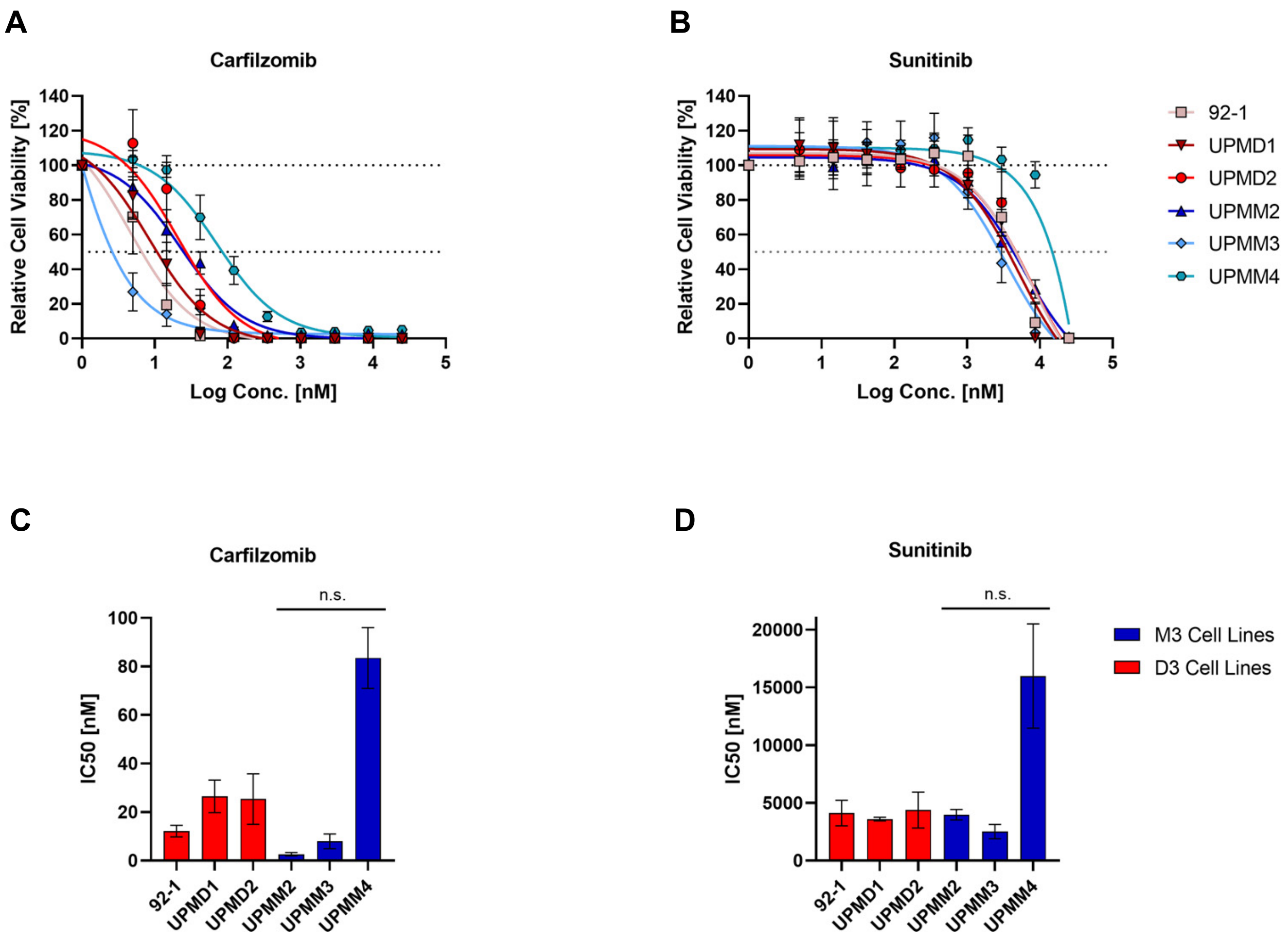

Figure S1. MEKi resistance pattern of M3 cell lines is not recapitulated in experiments with other compounds. Dose-response curves of uveal melanoma cell lines with M3 (blue symbols) and D3 (red symbols) that were treated with carfilzomib (**A**) or sunitinib (**B**) at different concentrations (from 5 nM to 25 µM) for 5 days and IC50 values for each cell line (**C, D**). Cell viability is expressed as normalized values in percentage to DMSO controls. Summarized results of three independent experiments are shown. Data are average ± SD.

BAP1

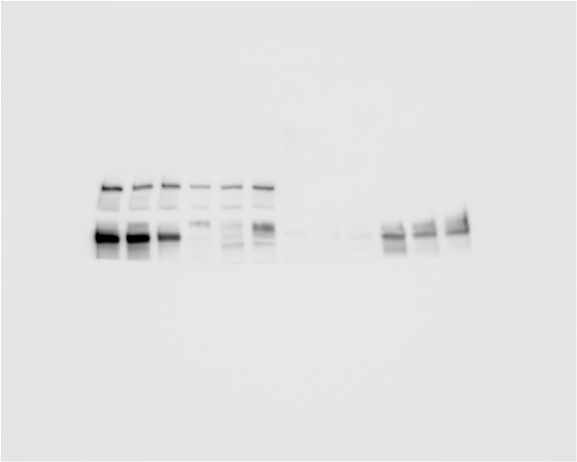

ß-Actin

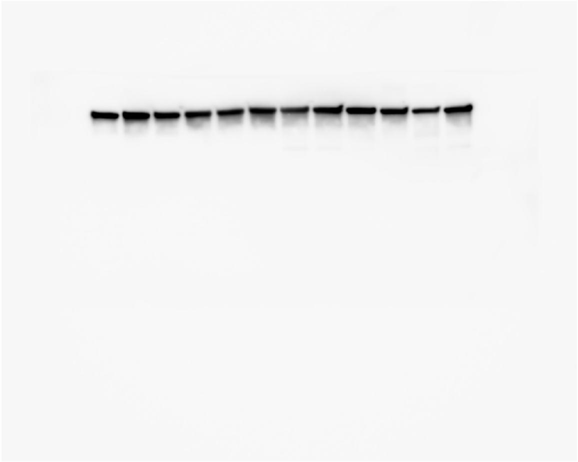

Figure S2. Uncropped western blots for Fig. 2. Original pictures of the western blots shown in Fig. 2.

BAP1

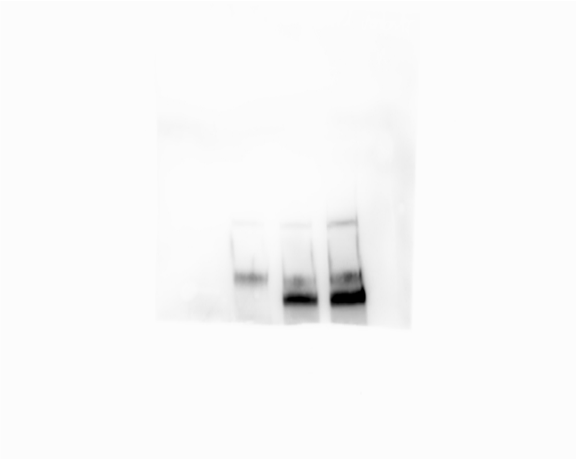

ß-Actin

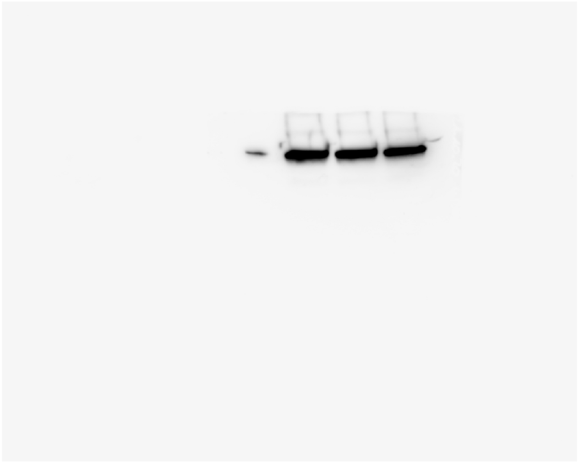

Figure S3. Uncropped western blots for Fig. 3. Original pictures of the western blots shown in Fig. 3.

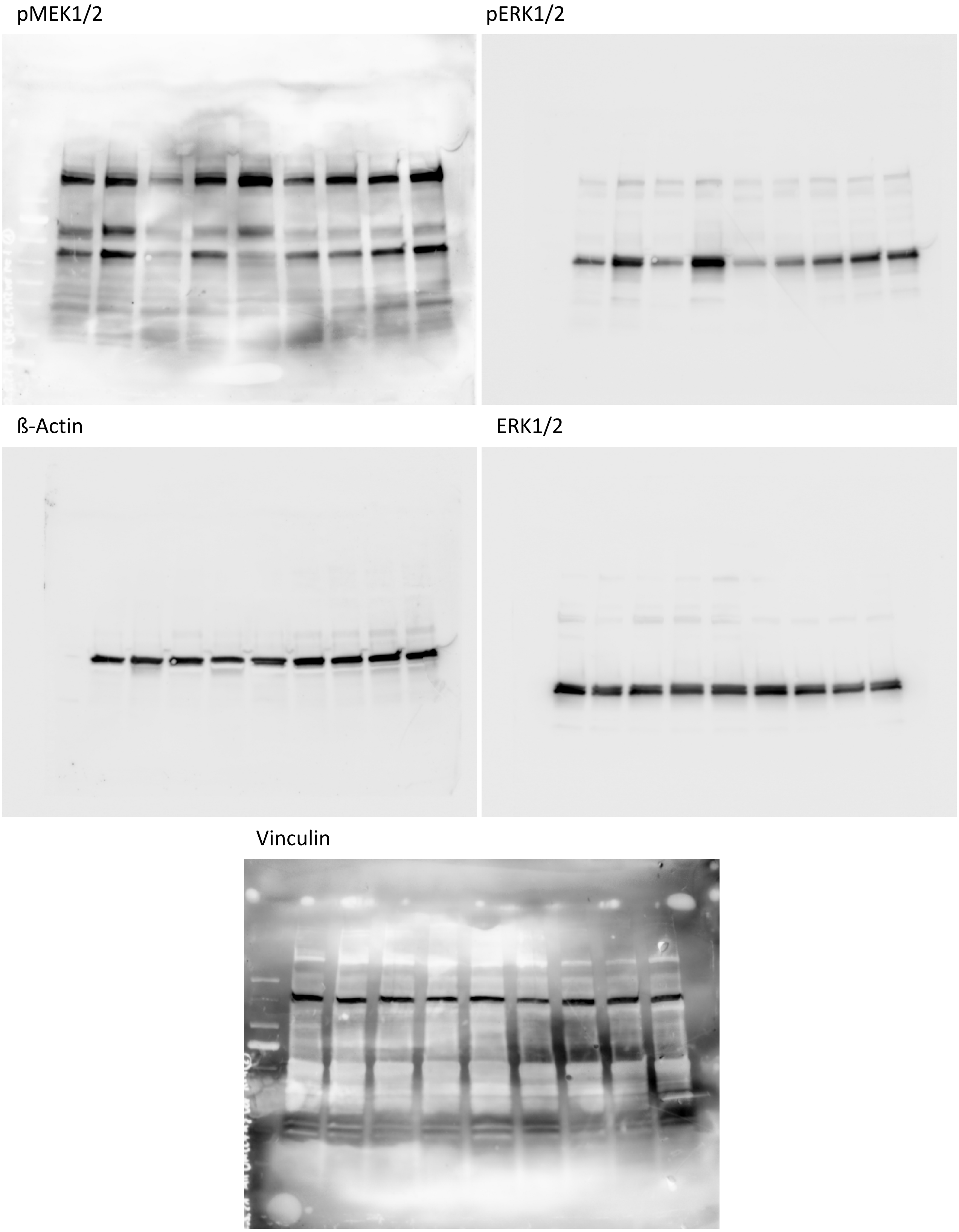

Figure S4. Uncropped western blots for Fig. 5A. Original pictures of the western blots shown in Fig. 5A.

pMEK1/2

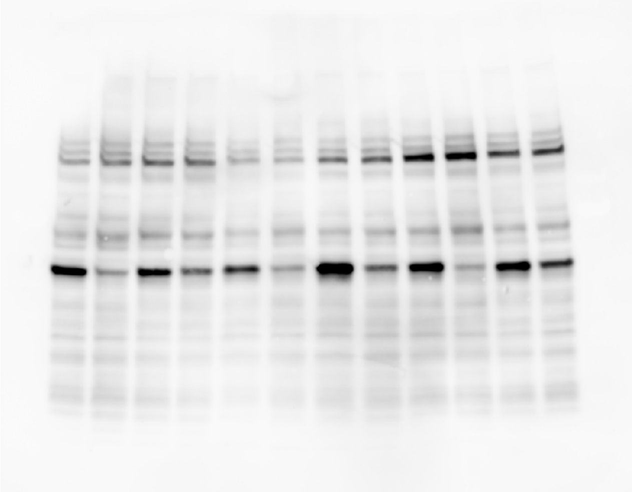

pERK1/2

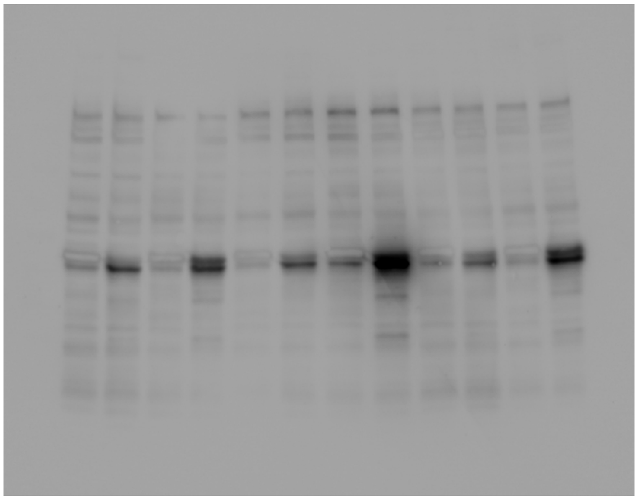

ß-Actin

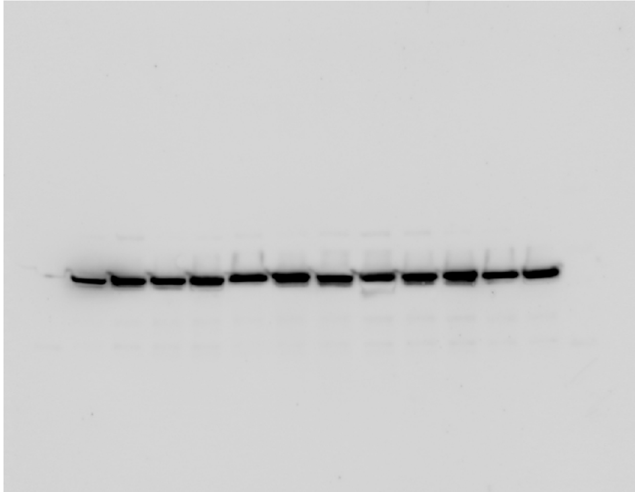

ERK1/2

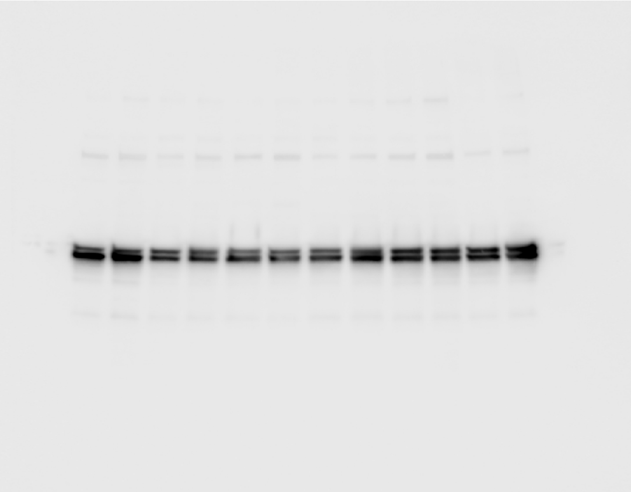

Vinculin

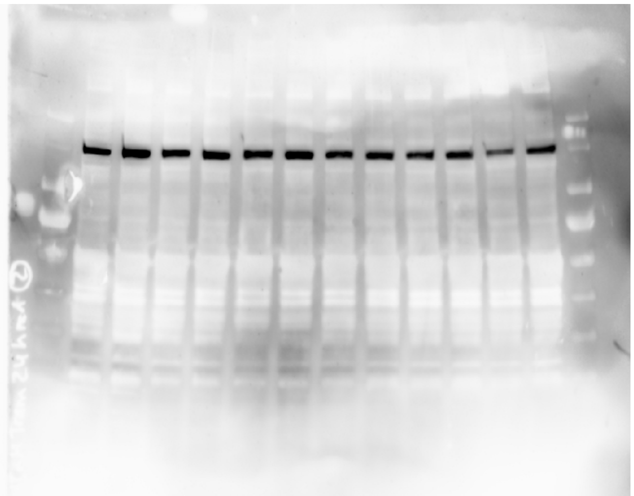

Figure S5. Uncropped western blots for Fig. 5B. Original pictures of the western blots shown in Fig. 5B.

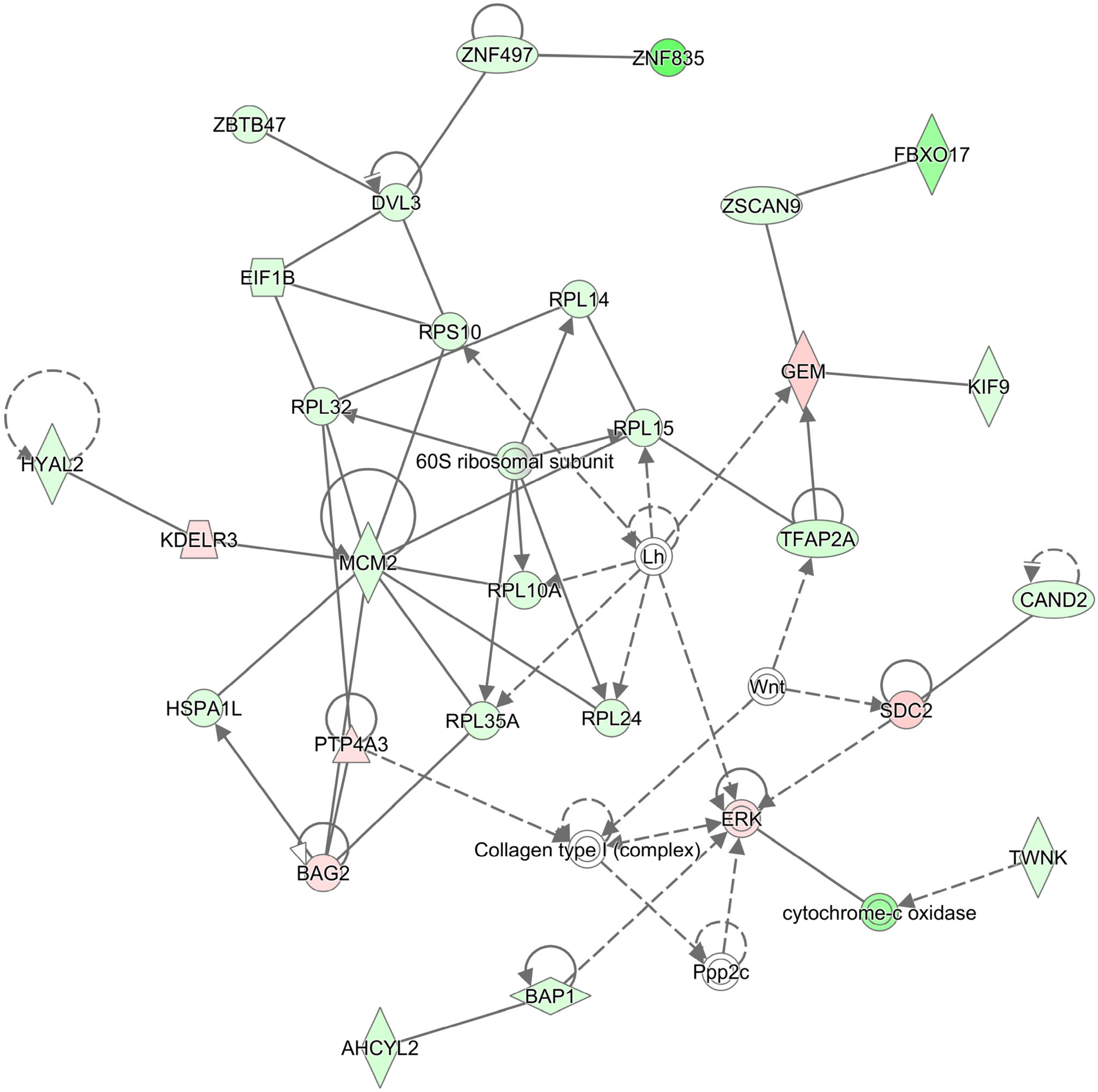

Figure S6. BAP1 regulates ERK indirectly. Network analysis of uveal melanoma TCGA gene expression data (*n*=74) comparing *BAP1*-mutant *vs. BAP1*-wild type tumor samples using Ingenuity Pathway Analysis (IPA) reveals an indirect interaction between BAP1 and ERK*.*

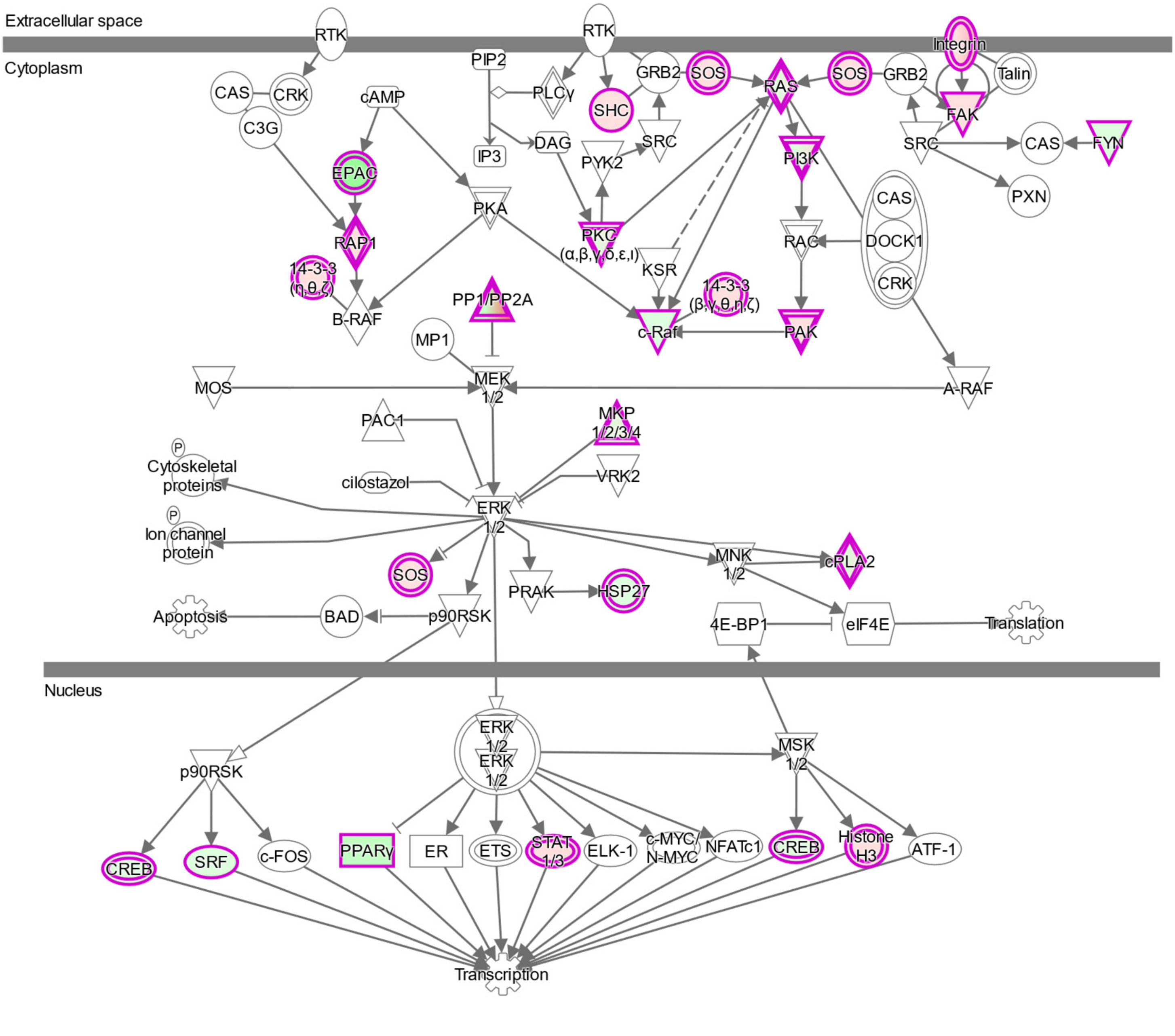

Figure S7. ERK/MAPK Signaling. Ingenuity Pathway Analysis shows upregulated genes in shades of red and downregulated genes in shades of green.

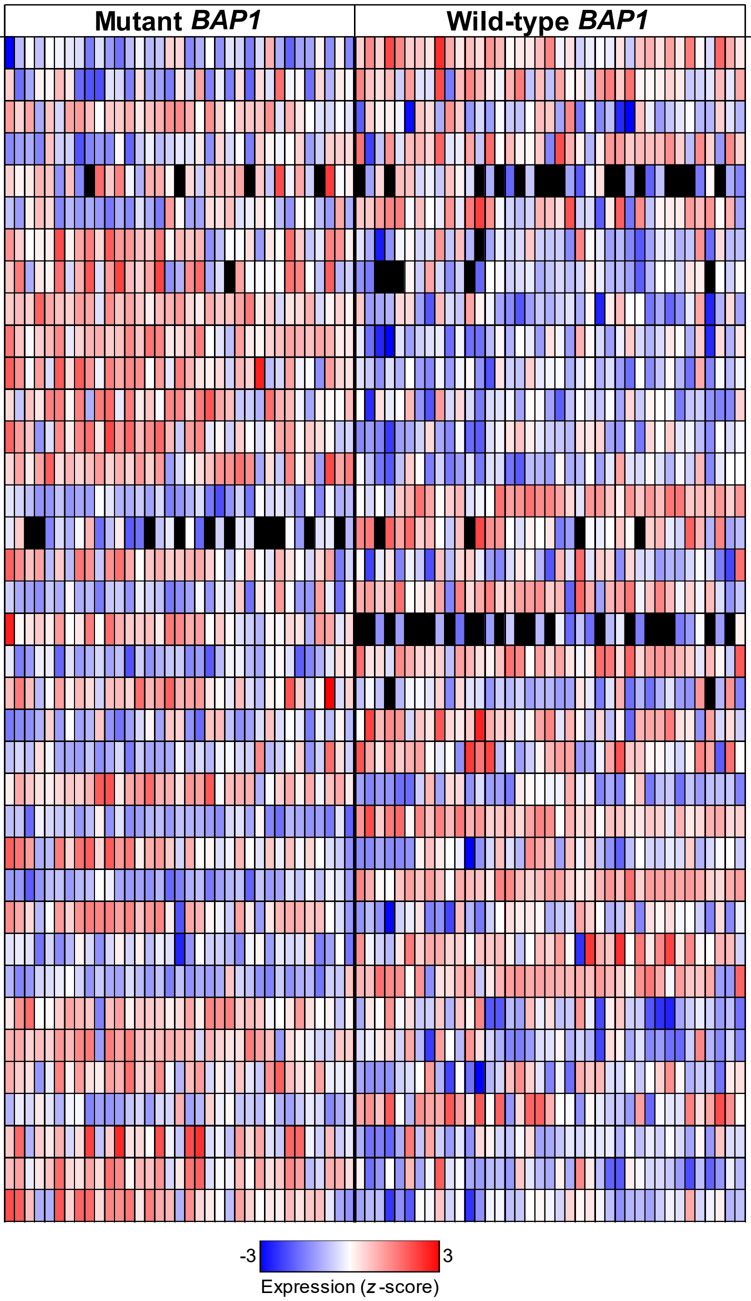

Gene	*p*	*FDR q*	FC
CREB3L4	1E-10	3E-9	-1.6
DUSP4	9E-5	5E-4	-1.8
DUSP9	1E-5	1E-4	1.7
FYN	2E-5	1E-4	-1.9
HIST2H3C	9E-5	6E-4	2.3
HSPB1	7E-5	4E-4	-1.8
ITGA2	1E-6	1E-5	2.9
ITGA4	2E-5	1E-4	6.8
ITGA5	1E-9	3E-8	2.4
ITGA6	5E-10	1E-8	2.5
ITGB3	3E-9	7E-8	4.0
PAK6	7E-7	7E-6	1.8
PIK3C2A	1E-7	2E-6	3.4
PIK3R3	5E-10	1E-8	3.0
PIK3R4	2E-18	5E-16	-2.3
PLA2G1B	8E-5	5E-4	-3.6
PLA2G4C	1E-4	7E-4	1.7
PPARG	5E-8	7E-7	-5.3
PPP1R14C	9E-10	2E-8	9.9
PPP1R3C	6E-15	6E-13	-4.7
PPP2R2B	8E-8	1E-6	14.1
PPP2R3B	2E-6	2E-5	-1.9
PPP2R5B	5E-5	3E-4	-1.7
PRKCA	1E-12	7E-11	2.9
PRKCD	1E-22	1E-19	-2.7
PTK2	4E-5	3E-4	1.6
RAF1	2E-27	5E-24	-2.1
RAP1B	7E-6	6E-5	1.5
RAP2B	1E-8	3E-7	-1.6
RAPGEF3	4E-15	5E-13	-8.9
RASD2	3E-6	3E-5	2.0
SHC1	7E-8	1E-6	1.6
SOS1	2E-4	1E-3	1.5
SRF	6E-7	7E-6	-1.5
STAT1	1E-5	8E-5	3.2
STAT3	8E-9	1E-7	1.6
YWHAZ	4E-7	5E-6	1.7

Figure S8. ERK/MAPK signaling pathway genes in *BAP1*-mutant and *BAP1*-wild type UM tumor samples. Uveal melanoma TCGA data (*n*=74) was analyzed and grouped in *BAP1*-mutant and *BAP1*-wild type tumor samples. The genes of the ERK/MAPK signaling pathway that were differentially expressed after a false discovery rate (FDR) corrected *p* value below 0.001 and a fold change (FC) higher than 1.5 are shown.

Supplementary Table S1. List of primers.

Primer name	Sequence 5’-3’
EIF2A_F	GACCCCAACCATACAAGGTGG
EIF2A_R	TTCTCCATAGTAGGAAGCTCCTG
EIF4A2_F	GAAGCCTTCCGCTATTCAGCA
EIF4A2_R	CTTGGGTCTCCTTGAACTCAATC
PPIB_F	GAGGAAAGAGCATCTACGGTG
PPIB_R	GCTTCTCCACCTCGATCTTG
RAF1_F	TGTTCCCCTCACAACACACAA
RAF1_R	CTGGGACTCCACTATCACCAATA
RPS12_F	TGCTGGAGGTGTAATGGACG
RPS12_R	GGCGCTTGTCTAAGGCTTTG
WARS_F	AAAGGCATTTTCGGCTTCACT
WARS_R	ATGGCACATGGGATAAGGCAC
